# Robotic Versus Laparoscopic Surgery for Colorectal Endometriosis: A Systematic Review and Meta-Analysis of Comparative Observational Cohorts

**DOI:** 10.7759/cureus.117578

**Published:** 2026-10-07

**Authors:** Zoya Shaikh, Mohammed Razeen Shaikh

**Affiliations:** 1 Intensive Care Medicine, Guy's and St Thomas' NHS Foundation Trust, London, GBR; 2 General Surgery, Milton Keynes University Hospital, Milton Keynes, GBR

**Keywords:** endometriosis surgery, general and colorectal surgeon, laparoscopic colon resection, robotic, robotic surgical procedures

## Abstract

This review compares perioperative and selected postoperative outcomes between robotic-assisted surgery and conventional laparoscopy in females undergoing surgical treatment for colorectal or rectosigmoid endometriosis. This systematic review and meta-analysis followed the PRISMA 2020 guidelines. PubMed, Web of Science, Embase, Scopus, Cumulative Index to Nursing and Allied Health Literature (CINAHL), and African Journals Online (AJOL) were searched from January 2020 to August 2026 for comparative observational studies evaluating robotic-assisted versus conventional laparoscopic surgery specifically for colorectal or rectosigmoid endometriosis. Manual bibliographic searches of the reference lists of relevant articles were also conducted to identify additional eligible studies. Studies involving general, superficial, ovarian, or deep endometriosis without separately identifiable colorectal disease were excluded. Study selection, data extraction, and risk-of-bias assessment were performed independently by two reviewers, with disagreements resolved by discussion. Risk of bias was assessed using the Newcastle-Ottawa Scale (NOS), and random-effects meta-analysis was undertaken only for outcomes with sufficiently comparable and non-duplicated data. Eight comparative observational reports met the eligibility criteria. Across studies reporting operative duration, robotic-assisted surgery was generally associated with longer operative time than conventional laparoscopy, although the magnitude and statistical significance of this difference varied between cohorts. Estimated blood loss, overall postoperative complications, conversion to open surgery, and most other perioperative outcomes were broadly comparable between approaches. Length of hospital stay showed inconsistent findings across studies, while selected reports suggested possible differences in voiding dysfunction, reoperation, recurrence, postoperative opioid use, and other longer-term outcomes. Considerable clinical and methodological heterogeneity was present because of differences in colorectal lesion complexity, extent of bowel surgery, associated pelvic procedures, institutional experience, follow-up duration, and outcome definitions. Potential overlap between several reports from the same specialist centre was addressed at the outcome level to avoid duplicate contribution of participants to pooled estimates. Overall, robotic-assisted surgery appears to be a feasible alternative to conventional laparoscopy for colorectal endometriosis, but current evidence does not demonstrate a consistent perioperative superiority of either approach. Because all available evidence is observational and susceptible to selection bias, residual confounding, temporal effects, and cohort overlap, causal conclusions cannot be drawn. Further prospective, multicentre, independently recruited comparative studies with standardized perioperative and long-term outcome reporting are required.

## Introduction and background

Robotic surgery has emerged as an increasingly used minimally invasive approach for complex endometriosis surgery, particularly when disease involves anatomically restricted areas of the pelvis. Endometriosis affects approximately 10% of women of reproductive age and is an important cause of chronic pelvic pain, infertility, and impaired quality of life [1]. The disease is characterized by endometrial-like glands and stroma outside the uterine cavity, accompanied by inflammatory, fibrotic, neuroangiogenic, and hormonal processes that contribute to progressive tissue distortion and organ involvement [2]. Among its most technically challenging forms is colorectal endometriosis, in which deep lesions involve the rectum, rectosigmoid junction, sigmoid colon, or adjacent bowel and may require advanced pelvic dissection or bowel resection.

Colorectal involvement is an important form of deep endometriosis because surgery must balance adequate disease excision against preservation of bowel, urinary, neurological, reproductive, and sexual function. Depending on lesion size, depth of bowel-wall infiltration, circumference involved, multifocality, and distance from the anal verge, surgical treatment may consist of rectal shaving, discoid excision, or segmental colorectal resection. These procedures carry recognized risks, including anastomotic leakage, rectovaginal fistula, pelvic abscess, haemorrhage, bowel stenosis, urinary dysfunction, and the possible need for temporary or permanent stoma formation [3]. For this reason, colorectal endometriosis surgery is commonly performed in specialist referral centres with experience in advanced minimally invasive and multidisciplinary pelvic surgery.

Conventional laparoscopy has long represented the principal minimally invasive approach for colorectal endometriosis. Robotic-assisted surgery has subsequently been introduced as an alternative, offering potential technical advantages such as three-dimensional magnified visualization, articulated wristed instruments, tremor filtration, improved surgeon ergonomics, and greater instrument dexterity within the confined deep pelvis. Whether these technical advantages translate into clinically meaningful benefits, however, remains uncertain. A systematic review and meta-analysis comparing robotic and conventional laparoscopic endometriosis surgery found no significant differences in blood loss, hospital stay, or complication rates, although robotic procedures tended to require longer operative time [4]. A later systematic review similarly found no demonstrable overall clinical advantage of robotic-assisted laparoscopy over conventional laparoscopy in endometriosis surgery [5].

The applicability of these broader findings to colorectal disease is uncertain because many earlier reviews combined superficial, ovarian, urinary, deep pelvic, and bowel endometriosis despite substantial differences in operative complexity [5]. A colorectal-specific systematic review and meta-analysis subsequently reported longer operative duration with robotic-assisted surgery but no significant differences in estimated blood loss, hospital stay, conversion to laparotomy, or overall postoperative complications compared with conventional laparoscopy [6]. Similarly, reviews addressing deep endometriosis more broadly, including bowel disease, reported that robotic surgery was a safe and feasible alternative with longer operative times, while hospital-stay findings differed between reviews [7,8]. These findings indicate that the comparative value of the two platforms remains uncertain, particularly when differences in bowel procedure, lesion severity, surgeon experience, and institutional practice are considered [9,10].

This systematic review and meta-analysis was therefore undertaken to update and synthesize comparative observational evidence involving women undergoing robotic-assisted versus conventional laparoscopic surgery specifically for colorectal or rectosigmoid endometriosis. Eight eligible comparative observational reports [11-18] were identified across the updated review period. Unlike broader reviews incorporating heterogeneous forms of endometriosis, the present review was restricted to studies in which colorectal involvement constituted the defined surgical population and both robotic-assisted and conventional laparoscopic groups were directly compared. Particular attention was given to potential overlap among publications originating from the same specialist centre and to differences in colorectal lesion complexity and procedure type.

Although a colorectal-specific meta-analysis was published in 2025 [6], important evidence has continued to emerge, including newer comparative observational studies. The present review was therefore undertaken to provide an updated synthesis of the available comparative evidence, with particular attention to perioperative outcomes and the clinical outcomes reported across contemporary studies. We restricted the search to publications from 2020 onward to focus on contemporary surgical practice and the recent expansion of comparative evidence on robotic-assisted surgery for colorectal endometriosis. This period also captures the studies published after earlier systematic reviews and reflects the more recent adoption and evaluation of robotic techniques.

Eligibility was restricted to direct robotic-versus-laparoscopic comparisons involving colorectal or rectosigmoid endometriosis. Studies without separately identifiable bowel involvement were excluded to maintain a strictly colorectal evidence base.

## Review

Methodology

Eight comparative observational reports were included in this systematic review and meta-analysis [11-18]. All directly compared robotic-assisted surgery with conventional laparoscopy in women undergoing surgical treatment for colorectal or rectosigmoid endometriosis. Studies of general, superficial, ovarian, urinary, or deep endometriosis were excluded unless a clearly identifiable colorectal population was reported separately. Not every report contributed to every pooled outcome because outcome definitions, statistical reporting, follow-up duration, and availability of extractable data differed between studies. Potential overlap among reports from the same specialist centre was specifically considered before quantitative pooling.

Search Strategy

A systematic literature search was performed in PubMed, Web of Science, Embase, Scopus, Cumulative Index to Nursing and Allied Health Literature (CINAHL), and African Journals Online (AJOL). Reference lists of relevant systematic reviews and eligible primary studies were also checked manually to identify additional comparative reports. The search covered publications from January 2020 to August 2026, with the final search update performed on 31st August 2026. Searches were limited to human studies published in English. Search syntax was adapted to each database, and its own subject headings were used where available.

The PubMed search was: ("colorectal endometriosis"[tiab] OR "rectosigmoid endometriosis"[tiab] OR "rectal endometriosis"[tiab] OR "bowel endometriosis"[tiab] OR "deep infiltrating endometriosis"[tiab] OR (Endometriosis[MeSH] AND (colorectal[tiab] OR rectal[tiab] OR rectosigmoid[tiab] OR bowel[tiab]))) AND ("robotic surgery"[tiab] OR "robot-assisted"[tiab] OR "robotic-assisted"[tiab] OR "Robotic Surgical Procedures"[MeSH]) AND (laparoscop*[tiab] OR Laparoscopy[MeSH]), with filters applied for 2020/01/01 to 2026/08/31, humans, and English.

The Scopus search was: TITLE-ABS-KEY(("colorectal endometriosis" OR "rectosigmoid endometriosis" OR "rectal endometriosis" OR "bowel endometriosis" OR "deep infiltrating endometriosis") AND ("robotic surgery" OR "robot-assisted" OR "robotic-assisted") AND laparoscop*) AND PUBYEAR > 2019 AND PUBYEAR < 2027 AND LANGUAGE(English).

The Web of Science search was: TS=(("colorectal endometriosis" OR "rectosigmoid endometriosis" OR "rectal endometriosis" OR "bowel endometriosis" OR "deep infiltrating endometriosis") AND ("robotic surgery" OR "robot-assisted" OR "robotic-assisted") AND laparoscop*), limited to 2020-2026 and English.

The Embase search was: ('endometriosis'/exp OR 'colorectal endometriosis' OR 'rectosigmoid endometriosis' OR 'bowel endometriosis') AND ('robotic surgery'/exp OR 'robot-assisted' OR 'robotic-assisted') AND ('laparoscopy'/exp OR laparoscop*), limited to 2020-2026, humans, and English.

The CINAHL search was: (MH "Endometriosis" OR "colorectal endometriosis" OR "rectosigmoid endometriosis" OR "bowel endometriosis") AND (MH "Robotic Surgery" OR "robot-assisted" OR "robotic-assisted") AND (MH "Laparoscopy" OR laparoscop*), limited to 2020-2026 and English.

AJOL does not support Boolean searching, so it was searched separately using the phrases "colorectal endometriosis robotic," "rectal endometriosis laparoscopy," and "bowel endometriosis robotic surgery," and the results were screened manually.

Finally, the reference lists of the included studies and of relevant systematic reviews were hand-searched to identify anything missed by database searches.

Study Selection

Studies were eligible if they included women undergoing surgery for confirmed colorectal, rectal, or rectosigmoid endometriosis and directly compared robotic-assisted surgery with conventional laparoscopic surgery within the same study. Eligible designs included prospective cohort studies, retrospective comparative cohorts, retrospective analyses of prospectively collected data, and propensity-matched observational studies. Studies had to report at least one relevant perioperative, postoperative, or longer-term clinical outcome.

Studies were excluded if they evaluated general endometriosis without separately extractable colorectal data, involved only superficial or ovarian disease, or focused mainly on non-bowel deep endometriosis. Single-arm robotic studies, single-arm laparoscopic series, case reports, conference abstracts, editorials, letters, reviews, previous meta-analyses, and studies without a clearly identifiable conventional laparoscopic comparator were excluded.

Titles and abstracts were screened independently by two reviewers against the eligibility criteria. The full texts of potentially eligible articles were then retrieved and assessed independently by the same two reviewers. Disagreements at either stage were resolved by discussion. Eight comparative observational reports ultimately met the inclusion criteria [11-18]. Table 1 summarizes the PICOST (Population, Intervention, Comparator, Outcomes, Study design, and Time) eligibility framework [19] used for study selection.

**Table 1:** PICOST criteria used for eligibility and study selection in the systematic review and meta-analysis. PICOST: Population, Intervention, Comparator, Outcomes, Study design, and Time; RAS: robotic-assisted surgery; LPS: conventional laparoscopic surgery.

PICOST component	Eligibility criteria applied in this review
Population (P)	Women undergoing surgical treatment for confirmed colorectal, rectal, or rectosigmoid endometriosis. Studies of general, superficial, ovarian, urinary, or deep endometriosis were eligible only when the colorectal population was clearly identifiable and separately reported.
Intervention/Exposure (I)	Robotic-assisted laparoscopic surgery (RAS) for colorectal or rectosigmoid endometriosis, including rectal shaving, discoid excision, segmental colorectal resection, colectomy, or other clearly defined minimally invasive colorectal procedures.
Comparator (C)	Conventional laparoscopic surgery (LPS) performed for the same colorectal or rectosigmoid endometriosis indication within the same comparative study.
Outcomes (O)	Primary outcomes: operative time, estimated blood loss, length of hospital stay, and overall postoperative complications. Secondary outcomes: intraoperative complications, major complications, conversion to open surgery, anastomotic leakage or fistula, reoperation or surgical revision, readmission or rehospitalization, voiding dysfunction, postoperative pain, analgesic or opioid use, recurrence, pregnancy/fertility-related outcomes, and other relevant postoperative or long-term clinical outcomes.
Study design (S)	Comparative observational studies, including prospective cohorts, retrospective cohorts, retrospective analyses of prospectively collected data, and propensity-matched comparative observational studies. Single-arm case series, case reports, conference abstracts, reviews, meta-analyses, editorials, letters, and studies without a conventional laparoscopic comparison group were excluded.
Time (T)	Studies published from January 2020 to August 2026 were eligible. The final eight included comparative observational reports were published between 2020 and 2026 [11-18].

Data Extraction and Critical Appraisal

Data were extracted from the full-text versions of the eight included reports [11-18] using a structured data-extraction form. Information collected included author and publication year, country, study centre or database, study design, recruitment period, number of patients in each surgical group, baseline characteristics, colorectal lesion characteristics, type of bowel procedure, associated pelvic procedures, follow-up duration, and reported outcomes.

Data extraction was performed independently by two reviewers using a pre-piloted form. The extracted data were then compared, and discrepancies were resolved by discussion. Where key data were unclear or missing, the original publication was rechecked.

The principal numerical outcomes extracted were operative time, estimated blood loss, length of hospital stay, and postoperative complications. Secondary outcomes included intraoperative complications, major postoperative complications, conversion to open surgery, reoperation or surgical revision, readmission or rehospitalization, anastomotic leakage or fistula, voiding dysfunction, postoperative pain, analgesic or opioid consumption, recurrence, pregnancy-related outcomes, and other longer-term clinical endpoints where reported.

Management of Potentially Overlapping Cohorts

Special attention was given to possible overlap between reports originating from the same specialist centre. Le Gac et al. [11], Ferrier et al. [13], Crestani et al. [14], and Crestani et al. [16] originated from the Tenon Hospital research stream and had partly overlapping recruitment periods. These publications were retained because they addressed different clinical questions and reported different outcomes, but they were not automatically treated as independent datasets in the same meta-analysis.

For each outcome, the most appropriate report was selected when substantial overlap was suspected. Preference was given to the larger, later, or more complete dataset for that specific endpoint. An overlapping publication could still contribute to a different outcome if it provided unique data not reported elsewhere. This approach reduced the risk of double-counting patients and artificially narrowing confidence intervals.

Outcome Variables

Four outcomes were defined as primary: operative time, estimated blood loss, length of hospital stay, and overall postoperative complications. Operative time was recorded in minutes using the closest measure of actual surgical duration reported by each study. When both total operating-room occupancy time and surgical time were available, surgical time was preferred. Estimated blood loss was recorded in millilitres, and hospital stay in days.

Overall postoperative complications were treated as a dichotomous outcome. Major complications were recorded separately when the original study used a recognized severity system such as Clavien-Dindo grade III or higher.

Secondary outcomes included intraoperative complications, conversion to laparotomy, anastomotic leakage or fistula, reoperation or revision, readmission or rehospitalization, voiding dysfunction, pain, analgesic or opioid use, recurrence, pregnancy-related outcomes, and other longer-term clinical outcomes reported by the included studies.

Risk of Bias Assessment

As all included studies were observational, methodological quality was assessed using the Newcastle-Ottawa Scale (NOS) for cohort studies [20]. The NOS evaluates three domains: selection of participants, comparability of treatment groups, and assessment of outcomes, with a maximum score of nine stars.

Each study was scored independently by two reviewers. Scores were compared, and disagreements were resolved by consensus or by consultation with a third reviewer.

Particular attention was given to non-random treatment allocation, disease complexity, lesion size and location, associated pelvic procedures, surgeon preference, institutional experience, calendar period, and extent of bowel resection. These factors were important because they could influence both the choice of surgical platform and the outcomes independently of the platform itself. Propensity matching or multivariable adjustment, when reported, was considered during the comparability assessment.

Data Synthesis and Statistical Analysis

Quantitative pooling was undertaken only when populations, outcome definitions, and statistical data were sufficiently comparable and when duplicate contribution from overlapping cohorts could reasonably be avoided. Continuous outcomes reported on the same scale were summarized using mean differences with 95% confidence intervals. Dichotomous outcomes were summarized using odds ratios or other appropriate comparative measures with 95% confidence intervals where sufficiently consistent data were available.

A random-effects model was used because the studies differed in disease severity, type of bowel surgery, surgeon experience, and follow-up. Mean differences (robotic minus laparoscopic) were calculated from the reported means, standard deviations, and group sizes. Studies were weighted by inverse variance. All analyses were performed using RevMan version 5.4 (Cochrane Collaboration, London, UK). Heterogeneity was assessed using the Q test and I² statistic, and I² above 50% was considered substantial. Studies reporting medians and interquartile ranges were not pooled and were summarized narratively.

A two-sided P-value below 0.05 was considered statistically significant. Non-significant findings were interpreted as absence of evidence for a statistically detectable difference and not as proof of equivalence.

When pooling was inappropriate because too few independent datasets were available, outcome definitions differed substantially, data were reported in incompatible statistical formats, or overlapping cohorts could not be separated safely, findings were summarized narratively. Funnel plots, Egger’s regression, and meta-regression were not considered reliable because fewer than 10 independent studies contributed to individual pooled outcomes.

Ethical Considerations

This systematic review used only previously published, anonymized, group-level data and involved no direct patient contact or access to individual medical records. Formal institutional ethical approval and individual informed consent were therefore not required. The review was conducted and reported according to the PRISMA 2020 recommendations [21].

Results

The included evidence comprised eight comparative observational reports [11-18] evaluating robotic-assisted surgery versus conventional laparoscopy in women undergoing surgical treatment specifically for colorectal, rectal, or rectosigmoid endometriosis. All available evidence was non-randomized; therefore, observed differences were interpreted as associations rather than causal treatment effects.

Figure 1 shows the PRISMA-based study selection process. A total of 574 records were identified through database searching. About 187 full-text articles were assessed for eligibility, with 179 excluded for predefined reasons, including non-colorectal populations, absence of a direct robotic-versus-laparoscopic comparison, or ineligible study design. Ultimately, eight observational studies met the eligibility criteria and were included in the systematic review.

**Figure 1:**
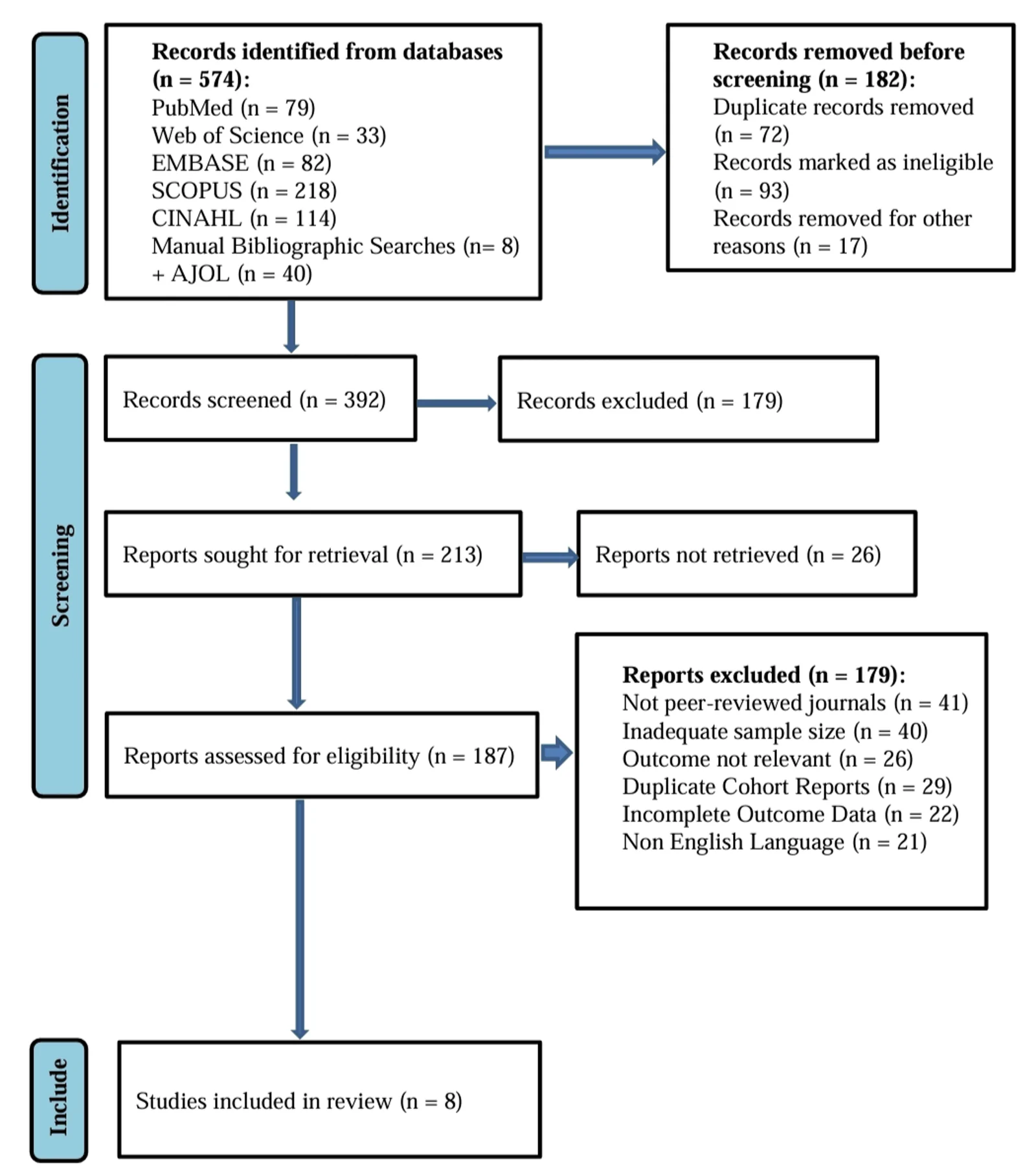
PRISMA 2020 flow diagram of the study selection process.

Systematic review

Operative Time

Longer operative duration with robotic surgery was observed in several earlier studies. Le Gac et al. [11] reported 221 ± 94 versus 163 ± 83 minutes (P=0.03), Ferrier et al. [13] reported 208 ± 90 versus 169 ± 81 minutes (P=0.01), and Crestani et al. [16] reported 216 ± 78 versus 190 ± 76 minutes (P=0.027). Raimondo et al. [12] also reported a numerically longer robotic operative time, although the difference was not statistically significant (207 ± 79 vs. 177 ± 63 minutes; P=0.171).

The 2026 Krentel study [17] showed the same numerical direction but no statistically significant difference: median skin-to-skin operative time was 225 minutes (IQR, 150-292) with robotic surgery versus 201 minutes (IQR, 146-251) with conventional laparoscopy (P=0.188), despite greater anatomical complexity in the robotic group. Because these data were reported as medians and interquartile ranges, they were not directly combined with the mean-and-standard-deviation data used in the exploratory mean-difference analysis.

Because Le Gac et al. [11], Ferrier et al. [13], and Crestani et al. [16] potentially contained overlapping Tenon Hospital patients, they were not pooled simultaneously. The later and more complete Crestani et al.'s dataset (2024) [16] was used to represent the Tenon stream, together with the independent Raimondo cohort [12]. Random-effects pooling of these two mean-based independent datasets produced a mean difference of 27.03 minutes, indicating longer operative time with robotic surgery (95% CI, 5.61-48.45; P=0.013). Heterogeneity was I²=0% (Q=0.03; df=1; P=0.87). Because only two datasets contributed to this pooled estimate, both the summary effect and heterogeneity statistic were regarded as exploratory.

Estimated Blood Loss

Le Gac et al. [11] reported mean estimated blood loss of 130 ± 86 mL with robotic surgery and 108 ± 99 mL with conventional laparoscopy. Raimondo et al. [12] reported 184 ± 214 versus 144 ± 101 mL, with no statistically significant difference. Ferrier et al. [13] and Crestani et al. [16] also reported no meaningful overall differences.

Krentel et al. [17] subsequently reported median blood loss of 100 mL in both groups (P=0.075), further supporting the absence of a clear difference, although their median-based data were not combined directly with the mean-based studies.

Using the two independent datasets with directly usable mean and standard-deviation data [11,12], exploratory random-effects pooling produced a mean difference of 25.94 mL (95% CI, −20.33 to 72.21; P=0.27). Heterogeneity was I²=0% (Q=0.10; df=1). The confidence interval crossed the null value and therefore provided no evidence of a statistically detectable difference in estimated blood loss.

Length of Hospital Stay

Hospital stay showed inconsistent findings. Le Gac et al. [11] reported a numerically longer stay after robotic surgery (8.0 ± 4.4 vs. 6.5 ± 2.6 days; P=0.18), while Raimondo et al. [12] similarly reported 8 ± 7 versus 6 ± 2 days (P=0.291). In contrast, Crestani et al. [16] found shorter mean hospital stay following robotic surgery (7 ± 4 vs. 8 ± 5 days; P=0.03).

Krentel et al. [17] also reported significantly shorter hospitalization after robotic surgery, with a median stay of five days (IQR, 4-6) versus six days (IQR, 4-7) (P=0.008). Because the study reported medians and interquartile ranges, it was retained in the narrative synthesis rather than combined directly with the mean-based pooled estimate.

Using the independent Raimondo [12] and Crestani (2024) [16] datasets, the exploratory random-effects pooled mean difference was 0.20 days (95% CI, −2.68 to 3.07; P=0.89). Heterogeneity was substantial (I²=66.8%; Q=3.01; df=1; P=0.083), reflecting opposite directions of effect. Because only two studies contributed, the heterogeneity estimate was interpreted cautiously. Overall, no consistent hospital-stay advantage could be established.

Overall Postoperative Complications

Overall complication rates were broadly similar between approaches. Le Gac et al. [11], Ferrier et al. [13], and Crestani et al. [16] found no significant overall differences. Raimondo et al. [12] reported postoperative complications in 18% of robotic patients and 5% of laparoscopic patients (P=0.345).

Krentel et al. [17] similarly reported overall complications in 10.2% of robotic patients and 12.9% of conventional laparoscopic patients (P=0.80), despite greater disease complexity in the robotic group.

A pooled overall complication estimate was not calculated because definitions differed, several Tenon reports potentially overlapped, and sufficiently homogeneous independent event data were not consistently available. Results were therefore synthesized narratively.

Intraoperative Complications

Intraoperative complications were uncommon. Le Gac et al. [11] found no statistically significant difference between groups. Ferrier et al. [13] reported fewer intraoperative complications in the robotic group among women undergoing discoid excision or segmental resection (2% vs. 14%; P=0.04). Crestani et al. [16] subsequently reported similar overall intraoperative complication rates.

Krentel et al. [17] likewise found no clear overall safety disadvantage with robotic surgery despite more complex disease. Because procedure definitions and populations differed, no quantitative synthesis was undertaken.

Major Complications

Major postoperative complications were uncommon, and no consistent platform-related difference was established. Le Gac et al. [11] reported grade III complications in 13% of robotic patients compared with none of the conventional laparoscopic patients (P=0.17). Crestani et al. [16] found broadly similar Clavien-Dindo complication profiles.

Krentel et al. [17] reported severe complications classified as Clavien-Dindo grade ≥IIIa in 1.7% of robotic patients and 6.9% of laparoscopic patients; the difference was not statistically significant (P=0.26). No grade IV or V complications occurred.

Conversion to Open Surgery

Conversion to laparotomy was rare. Raimondo et al. [12] reported one robotic conversion following an internal iliac vein injury and none after conventional laparoscopy. Krentel et al. [17] similarly reported one robotic conversion (1.7%) and none among 101 laparoscopic procedures (P=0.37).

Elhadi et al. [18] excluded patients converted to open surgery and therefore did not provide an unbiased comparison of conversion risk. Because events remained very uncommon and reporting differed between studies, conversion was not quantitatively pooled.

Reoperation/Surgical Revision

Ferrier et al. [13] found no significant difference in surgical revision between approaches. Krentel et al. [17] reported reoperation in 1.7% of robotic patients compared with 6.9% of laparoscopic patients, although the difference was not statistically significant (P=0.26).

Other reports used differing definitions of reintervention and serious complications. Therefore, no pooled estimate was calculated, and the available evidence did not establish a consistent difference in reoperation risk.

Readmission/Rehospitalization

Ferrier et al. [13] reported no significant difference in rehospitalization. Krentel et al. [17] similarly found comparable early readmission rates (6.8% vs. 5.9%; P=1.00).

Elhadi et al. [18] provided longer-term database-based information and reported fewer emergency readmissions in the robotic cohort at five years (26.7% vs. 35.1%). Because the study evaluated a different time horizon and outcome definition, these data were not combined with early postoperative readmission outcomes.

Anastomotic Leakage or Fistula

Volodarsky-Perel et al. [15] found that robotic versus laparoscopic surgery was not independently associated with anastomotic leakage or fistula after multivariable adjustment (adjusted odds ratio (aOR), 1.2; 95% CI, 0.3-4.0).

Krentel et al. [17] reported no anastomotic or suture leakage among 59 robotic procedures and two events among 101 laparoscopic procedures (0% vs. 2.0%; P=0.55). Both leakage cases required temporary stoma formation. Event numbers and procedural definitions were insufficient for reliable quantitative pooling.

Voiding Dysfunction

Voiding dysfunction showed variable results. Le Gac et al. [11] and Ferrier et al. [13] reported no significant overall differences. Volodarsky-Perel et al. [15] found no independent association between surgical platform and bladder dysfunction after multivariable adjustment (aOR, 0.5; 95% CI, 0.1-1.8). Crestani et al. [16], however, reported significantly less persistent voiding dysfunction following robotic surgery (5% vs. 25%; P=0.01).

Krentel et al. [17] reported temporary voiding dysfunction in three patients following conventional laparoscopic segmental rectal resection. Elhadi et al. [18] found no significant longer-term differences in urinary dysfunction or urinary incontinence between propensity-matched groups. Differences in definitions, duration of follow-up, and surgical subgroups precluded pooling.

Postoperative Pain and Analgesic and Opioid Use

Crestani et al. [14] reported greater total tramadol consumption after robotic surgery than conventional laparoscopy (322 (222) vs. 242 (292) mg; P=0.04). However, major changes in analgesic prescribing across calendar years substantially confounded this comparison.

Raimondo et al. [12] reported improvement in pain and bowel symptoms after both approaches at 12 months. Elhadi et al. [18] found lower recorded long-term opioid utilization after robotic colectomy at three years (68.4% vs. 89.3%; RR, 0.77; 95% CI, 0.72-0.82), with a similar pattern at five years (68.0% vs. 90.3%). Conversely, no significant differences were observed for pelvic or perineal pain, dysmenorrhea, or dyspareunia. Because these outcomes represented substantially different constructs and time periods, they were summarized narratively.

Recurrence and Pregnancy-Related Outcomes

Long-term recurrence and pregnancy-related outcomes were principally reported by Elhadi et al. [18]. At three years, recorded pregnancy achievement was higher after robotic colectomy (7.2% vs. 2.9%; RR, 2.50; 95% CI, 1.36-4.59), whereas recorded recurrence was lower (4.3% vs. 20.1%; RR, 0.21; 95% CI, 0.14-0.34). At five years, pregnancy achievement remained numerically higher (7.8% vs. 3.3%) and recurrence lower (4.7% vs. 18.5%) in the robotic cohort.

These findings were considered hypothesis-generating rather than evidence of causal benefit, because the study was retrospective, fertility intention was unavailable, and residual differences in follow-up and unmeasured clinical characteristics could remain despite propensity-score matching.

Overall Synthesis

Across the eight comparative observational reports [11-18], robotic-assisted colorectal endometriosis surgery was generally associated with longer or numerically longer operative duration, although this difference was not statistically significant in all cohorts. Estimated blood loss and overall perioperative complication rates were generally comparable between platforms. Hospital stay was inconsistent across the earlier evidence but was shorter after robotic surgery in two more recent reports [16,17]. Selected studies suggested potentially favourable robotic associations for persistent voiding dysfunction [16], procedure-specific intraoperative complications [13], and selected longer-term outcomes [18], but these findings were not consistently reproduced across independent populations.

The updated evidence base remains limited by observational treatment allocation, residual confounding, differences in colorectal procedure and anatomical complexity, varying statistical formats and follow-up periods, and potential population overlap among several Tenon Hospital publications [11,13,14,16]. Accordingly, the findings support robotic surgery as a feasible alternative to conventional laparoscopy but do not establish causal superiority of either platform.

Table 2 summarizes the publication and methodological characteristics of the eight comparative observational reports included in the review. It presents country, journal, study design, sample size, recruitment period, and follow-up information. The evidence spans 2020-2026 and includes European single- and multicentre cohorts together with a large propensity-matched United States database analysis.

**Table 2:** General methodological and publication characteristics of comparative observational studies evaluating robotic-assisted versus conventional laparoscopic surgery for colorectal endometriosis. RAS: robotic-assisted surgery; LPS: conventional laparoscopic surgery. Sample sizes represent the participants included in each published comparative analysis. For Elhadi et al. [18], the numbers represent the final propensity-matched cohorts.

Author	Country	Journal	Study design	Sample size	Recruitment/Follow-up
Le Gac et al., 2020 [11]	France	J Gynecol Obstet Hum Reprod	Prospective comparative cohort	48; RAS 23, LPS 25	Mar-Sep 2019; short postoperative follow-up
Raimondo et al., 2021 [12]	Italy	Acta Obstet Gynecol Scand	Multicentre prospective observational cohort	44; RAS 22, LPS 22	Sep 2018-Sep 2019; 12 months
Ferrier et al., 2022 [13]	France	Int J Med Robot	Prospective comparative cohort	122; RAS 61, LPS 61	18-month recruitment period
Crestani et al., 2023 [14]	France	J Robot Surg	Retrospective comparative cohort	162; RAS 89, LPS 73	Feb 2019-Dec 2021; hospitalization outcomes
Volodarsky-Perel et al., 2023 [15]	France	Colorectal Dis	Retrospective cohort using prospectively collected data	548; RAS 97, LPS 451	Postoperative complication follow-up
Crestani et al., 2024 [16]	France	J Robot Surg	Retrospective comparative cohort	152; RAS 90, LPS 62	2019-2023; postoperative outcomes
Krentel et al., 2026 [17]	Germany	J Robot Surg	Retrospective single-centre sequential comparative cohort	160; RAS 59, LPS 101	2022-2025; perioperative outcomes
Elhadi et al., 2026 [18]	United States	J Robot Surg	Retrospective multicentre propensity-score-matched database cohort	974 matched; RAS 487, LPS 487	Oct 2013-Oct 2025; outcomes assessed at 3 and 5 years

Table 3 describes the colorectal disease populations and operative procedures represented across the eight reports. All studies specifically evaluated colorectal, rectal, rectosigmoid, or bowel deep endometriosis rather than mixed general endometriosis populations. Operative complexity ranged from shaving and mucosa-sparing excision to segmental resection and colectomy, contributing importantly to clinical heterogeneity.

**Table 3:** Disease characteristics and colorectal surgical procedures in the eight included comparative observational reports. RAS: robotic-assisted surgery; LPS: conventional laparoscopic surgery; ENZIAN C3: colorectal endometriotic involvement classified within compartment C with lesions >3 cm; CNR = complete mucosa-sparing nodular resection; SRR: segmental rectal resection.

Author	Defined study population	Robotic group	Laparoscopic group	Colorectal procedures/Characteristics	Important clinical feature
Le Gac et al., 2020 [11]	Colorectal endometriosis requiring surgery	23	25	Rectal shaving, discoid excision, segmental resection	Baseline characteristics generally similar; trend toward more associated urinary/digestive procedures with RAS
Raimondo et al., 2021 [12]	Rectosigmoid endometriosis	22	22	Surgical excision of rectosigmoid disease	More hysterectomies and bilateral uterosacral ligament excisions in RAS group; adjusted analysis performed
Ferrier et al., 2022 [13]	Colorectal endometriosis	61	61	Shaving, discoid excision, segmental resection	Equal group sizes; specific analysis of discoid/segmental procedures
Crestani et al., 2023 [14]	Colorectal endometriosis undergoing surgery	89	73	Shaving, discoid resection, segmental resection	Main focus was postoperative analgesic consumption
Volodarsky-Perel et al., 2023 [15]	Rectal endometriotic nodule >3 cm (ENZIAN C3)	97	451	Rectal shaving, disc excision, colorectal resection	Large cohort restricted to large rectal nodules
Crestani et al., 2024 [16]	Colorectal endometriosis requiring bowel-wall excision/resection	90	62	Discoid excision or segmental colorectal resection	Consecutive cohort restricted to more extensive colorectal procedures
Krentel et al., 2026 [17]	Histologically confirmed symptomatic colorectal endometriosis	59	101	Complete mucosa-sparing nodular resection or segmental rectal resection with anastomosis	RAS group had significantly greater Enzian-defined anatomical complexity, including more advanced rectal and multicompartmental disease
Elhadi et al., 2026 [18]	Adult women undergoing minimally invasive colectomy for colorectal deep infiltrating endometriosis	487	487	Robotic versus conventional laparoscopic colectomy	TriNetX network of 72 US healthcare organizations; 1:1 propensity matching; conversions to open surgery were excluded

Table 4 presents the NOS [20] assessment for the eight comparative observational reports. The NOS evaluates cohort selection, comparability of treatment groups, and outcome ascertainment. Methodological quality varied because of non-randomized treatment allocation, residual confounding, temporal changes in practice, differences in disease complexity, and limitations inherent to retrospective database-based analyses.

**Table 4:** Newcastle-Ottawa Scale assessment of methodological quality across the eight included comparative observational reports. NOS: Newcastle-Ottawa Scale. The selection domain has a maximum of four stars, comparability a maximum of two stars, and outcome a maximum of three stars, giving a maximum total of nine. In this review, scores ≥7 were categorized as higher methodological quality and scores <7 as moderate/lower quality.

Author	Selection/4	Comparability/2	Outcome/3	Total/9	Quality interpretation	Main methodological considerations
Le Gac et al., 2020 [11]	4	1	2	7	Higher quality	Prospective comparative cohort; same-source comparator; broadly similar baseline characteristics; limited adjustment and short postoperative follow-up
Raimondo et al., 2021 [12]	4	2	3	9	Higher quality	Multicentre prospective design; clearly defined rectosigmoid population; adjusted analysis; structured 12-month follow-up
Ferrier et al., 2022 [13]	4	1	2	7	Higher quality	Prospective design; equal group sizes; objective outcomes; limited formal adjustment for treatment-selection confounding
Crestani et al., 2023 [14]	3	1	2	6	Moderate quality	Retrospective design; marked calendar-year change in analgesic protocols; temporal confounding despite regression analysis
Volodarsky-Perel et al., 2023 [15]	4	2	2	8	Higher quality	Large defined rectal cohort; prospectively collected data; multivariable adjustment for major complication predictors
Crestani et al., 2024 [16]	4	1	2	7	Higher quality	Consecutive retrospective cohort; clearly defined colorectal procedures; objective outcomes; limited adjustment for residual confounding
Krentel et al., 2026 [17]	4	0	3	7	Higher quality*	Consecutive single-centre cohort with objective perioperative outcomes, but sequential non-randomized treatment allocation and substantially greater anatomical complexity in the RAS group without formal risk-adjusted comparison
Elhadi et al., 2026 [18]	4	2	2	8	Higher quality*	Large multicentre database cohort with 1:1 propensity matching and long-term outcomes; residual unmeasured confounding, database coding limitations, differential follow-up, unavailable fertility intention, and exclusion of conversions remain important limitations

Figure 2 presents overlap-aware quantitative comparisons of robotic-assisted surgery with conventional laparoscopy for colorectal endometriosis. Robotic surgery demonstrated longer operative time in the mean-based exploratory analysis, whereas estimated blood loss and hospital stay showed no statistically significant pooled differences. Krentel et al. [17] was synthesized narratively because key continuous outcomes were reported as medians and interquartile ranges, while Elhadi et al. [18] principally contributed longer-term outcomes that were not directly comparable with the perioperative datasets. Complications, conversion, anastomotic events, voiding dysfunction, analgesic or opioid use, recurrence, and pregnancy-related outcomes were therefore primarily summarized narratively.

**Figure 2:**
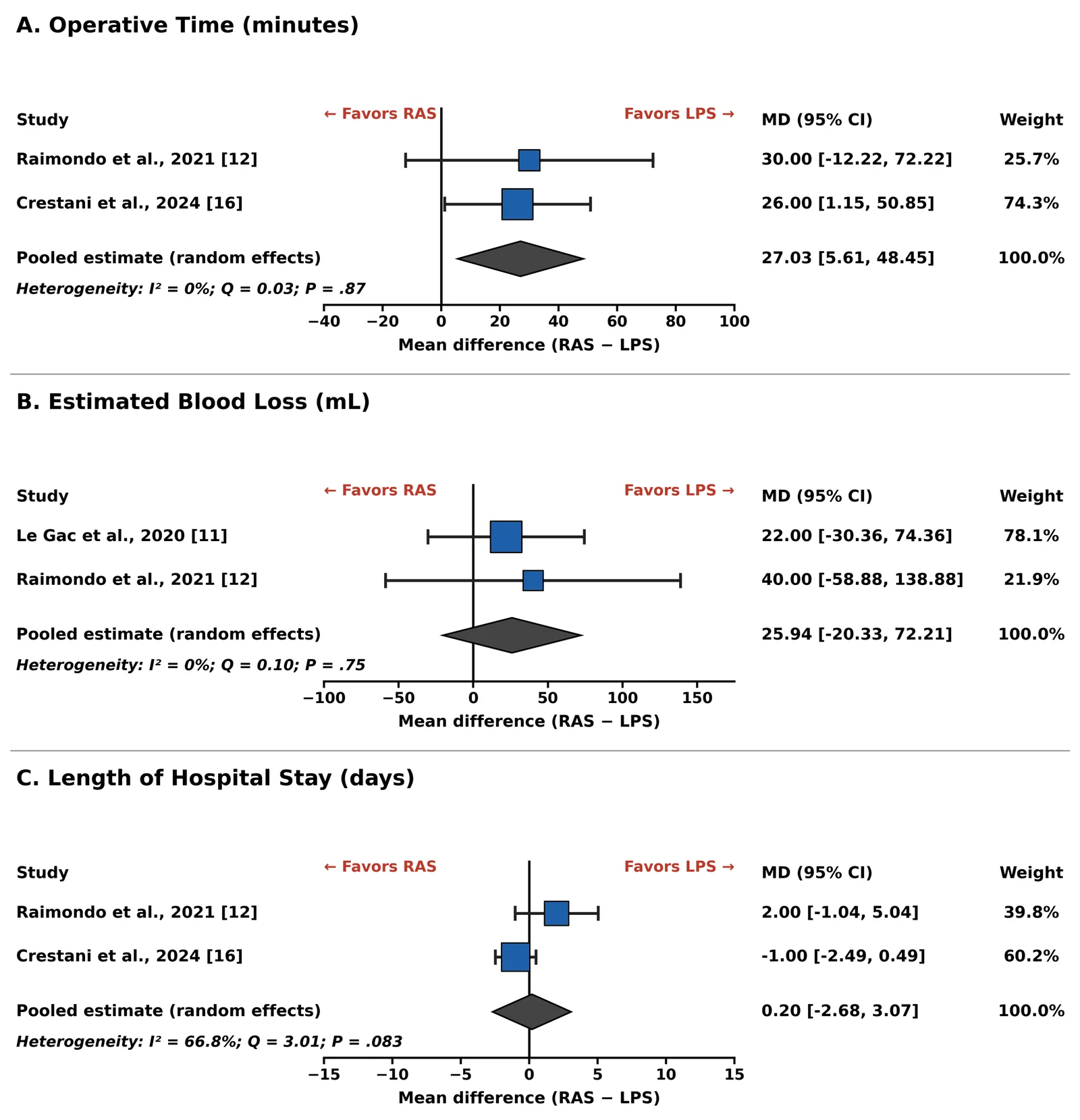
Forest plot of quantitative comparisons of robotic-assisted surgery versus conventional laparoscopy for colorectal endometriosis. Mean differences are robotic minus laparoscopic. RAS: robotic-assisted surgery; LPS: conventional laparoscopic surgery; MD: mean difference; CI: confidence interval. Random-effects model with inverse-variance weighting and the DerSimonian–Laird estimator.

Discussion

Comparison With Previous Literature

This systematic review and meta-analysis evaluated eight comparative observational reports [11-18] examining robotic-assisted versus conventional laparoscopic surgery specifically for colorectal or rectosigmoid endometriosis. The updated evidence generally showed longer or numerically longer operative duration with robotic surgery, whereas estimated blood loss and overall perioperative complication rates were broadly comparable between approaches. Hospital stay was inconsistent across earlier studies, although two more recent reports [16,17] found shorter hospitalization after robotic surgery. Selected studies also suggested potentially favourable robotic associations for intraoperative complications, persistent voiding dysfunction, recurrence, pregnancy-related outcomes, and long-term opioid use; however, these findings were not consistently reproduced across independent datasets and should not be interpreted as evidence of causal superiority.

The operative-time pattern remains broadly consistent with the earlier colorectal-specific meta-analysis, which reported a pooled weighted mean difference of 21.51 minutes longer with robotic surgery and no significant differences in blood loss, hospital stay, conversion, or overall complications [6]. The present review extends that evidence through 2026 and incorporates two additional comparative reports [17,18]. Krentel et al. [17] found numerically longer robotic operative time despite greater Enzian-defined anatomical complexity in the robotic group, while Elhadi et al. [18] expanded the evidence toward longer-term outcomes through propensity-matched US data.

Interpretation of safety outcomes is complicated by the heterogeneity of bowel surgery itself. Rectal shaving, discoid excision, and segmental colorectal resection differ substantially in operative complexity and postoperative risk. A comparative series of 364 women with rectosigmoid endometriosis reported more severe postoperative complications after segmental resection than after more conservative procedures [22]. A separate systematic analysis likewise found lower major-complication rates with conservative shaving or discoid excision than with segmental bowel resection, while emphasizing the limited quality and heterogeneity of the evidence [23]. These findings reinforce the importance of procedure type and disease severity as potential confounders in robotic-versus-laparoscopic comparisons. The lower procedure-specific intraoperative complication rate reported by Ferrier et al. [13] is therefore clinically interesting but cannot alone establish a platform advantage.

Postoperative urinary dysfunction illustrates this difficulty particularly well. Le Gac et al. [11] and Ferrier et al. [13] found no clear overall difference, Volodarsky-Perel et al. [15] found no independent association after multivariable adjustment, and Crestani et al. [16] reported less persistent dysfunction with robotic surgery. The 2026 evidence similarly did not establish a consistent platform effect. An updated systematic review of colorectal deep endometriosis found substantially less postoperative voiding dysfunction following rectal shaving than segmental resection, suggesting that extent of bowel and autonomic-nerve dissection may be more influential than surgical platform alone [24].

Taken together, the updated literature supports robotic surgery as a technically feasible alternative rather than a clearly superior treatment. Potential benefits may be most relevant in complex deep-pelvic disease, but the present observational evidence does not demonstrate a consistent reduction in blood loss, major complications, conversion, or other core perioperative outcomes.

Limitations of the Study

Several limitations remain. All eight reports were observational, so treatment allocation could have been influenced by surgeon preference, robotic availability, calendar period, lesion complexity, previous surgery, associated procedures, and anticipated technical difficulty. Krentel et al. [17], for example, included greater anatomical complexity in the robotic group, while propensity matching in Elhadi et al. [18] could address only measured confounders. Residual confounding therefore remains unavoidable.

The quantitative analyses also contained very few independent datasets. Operative time and hospital stay were pooled from two independent mean-based datasets, while the blood-loss analysis likewise included only two studies. Estimates of heterogeneity are therefore imprecise, and non-significant results cannot be interpreted as evidence of equivalence. In addition, Krentel et al. [17] reported several continuous outcomes as medians and interquartile ranges, and were therefore synthesized narratively rather than directly combined with mean-based estimates.

Outcome definitions varied considerably. Operative time, total operating-room time, complication severity, urinary dysfunction, pain, opioid exposure, recurrence, and fertility-related outcomes were not measured uniformly. Elhadi et al. [18] also assessed substantially longer-term outcomes than most earlier reports and excluded conversions to open surgery, limiting direct comparability. Publication bias could not be reliably assessed because fewer than 10 independent studies contributed to any pooled outcome.

Clinical Implications and Future Research

For clinical practice, the current evidence does not justify selecting robotic surgery solely on the expectation of lower perioperative morbidity. Conventional laparoscopy remains an established minimally invasive approach, while robotic assistance may be particularly useful in selected anatomically complex cases. Choice of platform should therefore incorporate disease extent, planned bowel procedure, surgeon experience, institutional expertise, and available resources.

Economic considerations are also important. A detailed public-sector cost analysis demonstrated substantial implementation, maintenance, consumable, and procedure-related expenditure associated with robotic programmes [25]. Where clinical outcomes are broadly comparable, these costs become particularly relevant in resource-limited health systems.

Future research should prioritize independent multicentre recruitment, transparent reporting of overlapping populations, standardized operative definitions, and adjustment for lesion complexity and surgical technique. Broader robotic-surgery evidence has shown substantial variability in study quality, outcome reporting, and transparency, including repeated publications derived from common underlying populations [26]. Future studies should clearly identify prior publication of participants and stratify results by shaving, discoid excision, and segmental resection.

Patient-centred outcomes should also be standardized. The international endometriosis core outcome set includes pain, improvement in the most troublesome symptom, quality of life, adverse events, satisfaction, and reproductive outcomes. For colorectal surgery, long-term bowel function, urinary function, recurrence, anastomotic morbidity, reoperation, fertility, and quality of life should additionally be reported consistently [27].

## Conclusions

This updated systematic review of eight comparative observational reports suggests that robotic-assisted colorectal endometriosis surgery is generally associated with longer or numerically longer operative duration, while estimated blood loss and overall perioperative safety are broadly comparable with conventional laparoscopy. Hospital stay and selected secondary outcomes showed inconsistent or study-specific differences.

Although newer studies extend the evidence toward more complex disease and longer-term outcomes, the literature remains limited by non-randomized treatment allocation, residual confounding, heterogeneous colorectal procedures, inconsistent outcome definitions, and overlap among several institutional reports. The available evidence therefore does not establish superiority or equivalence of either platform. Robotic surgery should presently be regarded as a feasible alternative, with selection individualized according to disease complexity, planned bowel procedure, surgical expertise, and institutional resources. Larger independent prospective multicentre studies with standardized outcomes are required.
